# Distribution and Redistribution of ^109^Cd and ^65^Zn in the Heavy Metal Hyperaccumulator *Solanum nigrum* L.: Influence of Cadmium and Zinc Concentrations in the Root Medium

**DOI:** 10.3390/plants8090340

**Published:** 2019-09-10

**Authors:** Urs Feller, Iwona Anders, Shuhe Wei

**Affiliations:** 1 Institute of Plant Sciences and Oeschger Centre for Climate Change Research, University of Bern, Altenbergrain 21, CH-3013 Bern, Switzerland; 2 Key Laboratory of Pollution Ecology and Environmental Engineering, Institute of Applied Ecology, Chinese Academy of Sciences, Shenyang 110016, China

**Keywords:** cadmium, zinc, concentration in medium, redistribution, xylem, phloem, root-to-shoot transfer, hyperaccumulator, bioremediation, *Solanum nigrum* L.

## Abstract

Heavy metal redistribution is relevant for the quality of edible crops and the suitability of hyperaccumulators for bioremediation. Root-to-shoot transfer via the xylem and redistribution in the aerial parts via the phloem differ between various heavy metals. In general, cadmium is more slowly released to the shoot than zinc (e.g., in wheat, bean, and lupin). However, rapid cadmium transport to the shoot was detected in the hyperaccumulator *Solanum nigrum* L. This is a key aspect in this article and might be important for bioremediation. The radionuclides ^109^Cd and ^65^Zn were used to investigate the respective influence of elevated cadmium or zinc in the root medium on the dynamics of the two heavy metals in *S. nigrum*. Although transport via the xylem to the leaves was similar for ^109^Cd and ^65^Zn, the further redistribution from older leaves to younger leaves, flowers, and fruits via the phloem was far less efficient for ^109^Cd than for ^65^Zn. Furthermore, the redistribution of ^109^Cd within the shoot was negatively influenced by increased cadmium (but not by increased zinc) concentrations in the nutrient medium. The redistribution of ^65^Zn in the shoot was selectively decreased by increased zinc concentrations (but generally not by cadmium).

## 1. Introduction

Heavy metal uptake, distribution, and redistribution processes on the whole plant level are important aspects for major crop plants [1,2,3,4], as well as for heavy metal hyperaccumulators [5,6,7,8]. Heavy metal contents in harvested products (e.g., cereal or soybean grains) are highly relevant for the quality of plant products for human or animal nutrition [9,10,11]. The acquisition and distribution of heavy metals are important aspects when considering hyperaccumulators for bioremediation purposes [7,8,12,13,14]. Besides the uptake into the roots of such plants, the root-to-shoot transfer and the distribution within the aerial plant parts are key aspects in this context, allowing efficient heavy metal removal from contaminated fields.

The acropetal transport with the transpiration stream in the xylem and the symplastic long-distance transport via the phloem are both important for the final distribution of the various heavy metals in a plant [8,15,16,17,18,19]. The distribution patterns in plants may differ considerably between various heavy metals and plant species. Cobalt is strongly retained in the root system [8,18,20]. In contrast, manganese is easily transported to transpiring leaves, but following this, is not efficiently redistributed within the shoot [16,18,20]. Nickel is very mobile in plants and directed to growing plant parts (root system, youngest shoot organs) [4,16,18,20]. In general, zinc is more rapidly released from the roots to the shoot than cadmium and is also then more easily redistributed within the shoot via the phloem [18,20]. The redistribution of a heavy metal may additionally be affected by environmental conditions, such as drought [21], light intensity [22], or nitrogen source [23,24], as well as by the availability of other heavy metals [25]. Cadmium and zinc contents are often both increased in polluted soils [26,27]. Cadmium/zinc interactions influencing the subcellular distribution and long-distance transport in crop plants were recently reported [23,24]. Cadmium uptake in wheat was found to be inhibited by zinc on a nutrient medium containing ammonium, while cadmium transport was stimulated by zinc [23]. Furthermore, the chemical form of the nitrogen source (ammonium or nitrate) modified cadmium/zinc interactions in these plants [23,24].

Several processes may influence the mobility of heavy metals on the whole plant level and these processes may be, to some extent, specific for certain elements. For some heavy metals, a considerable portion is usually retained in the root system. This may be caused by precipitation or ion exchange processes at the root surface or in the root apoplast before reaching the plasmalemma of root cells or by cellular/subcellular compartmentation after uptake into root cells [4,19]. Complexes with organic acids, nicotianamine, or phytochelatin may influence the root-to-shoot transfer [28,29,30]. All the processes mentioned may contribute to the discrimination between various heavy metals. In general, cobalt and, to a lesser extent, also cadmium, are rather strongly retained in the root system, while zinc and nickel are far more mobile [4,18,20]. 

*Solanum nigrum* L. grown on contaminated soil can accumulate large quantities of cadmium in the shoot, indicating that this species represents a cadmium hyperaccumulator and might be useful for the bioremediation of soils contaminated with this heavy metal [13]. *S. nigrum* tolerates high cadmium levels in the soil and enriches it in the shoot (13). The rapid transfer of cadmium and zinc to the shoot (an important property of a hyperaccumulator) was confirmed in a hydroponic culture [8]. In contrast to wheat, dwarf bean, or lupin, cadmium and zinc are rapidly transferred from the roots to the aerial plant parts of the hyperaccumulator *S. nigrum* [4,8,18,20]. Bean was extremely inefficient in the transfer of cadmium from the medium to the shoot, regardless of the cadmium concentration in the medium, while wheat was only slightly more efficient [25]. In wheat and bean, the zinc transfer to the shoot was somewhat better than for cadmium, but it was still far less efficient than in *S. nigrum* [25]. For the study reported here, the relative mobilities of the often co-occurring elements cadmium and zinc and possible interferences in the transport of the two elements are particularly interesting. It appears likely that xylem loading in the roots is a key process for rapid transport via the transpiration stream from the root system to the leaves [4,19]. Increased cadmium or zinc levels in the root medium only caused rather modest effects on the transport of ^109^Cd and ^65^Zn in bean and wheat [25]. Since *S. nigrum* differs considerably from wheat and bean in the accumulation and transport of cadmium and zinc on the whole plant level, the results obtained with the two crop plants mentioned cannot be simply transferred to this hyperaccumulator [8,18,20]. 

The aim of the work reported here was to identify the influence of an increased zinc or cadmium supply on the dynamics of these elements, with special reference to interactions between cadmium and zinc translocation in the hyperaccumulator *S. nigrum*. The radionuclides ^109^Cd and ^65^Zn allow sensitive and simultaneous detection in various plant parts with gamma spectrometry [8,15,16,18,20,21,22,25]. Besides the root-to-shoot transfer, the redistribution within the shoot from older leaves to younger leaves and finally to flowers/fruits can be addressed with the technique used. Additionally, visualization of the distribution/redistribution of ^65^Zn can be envisaged with autoradiography [18,20,21].

## 2. Results

**Figure 1:**
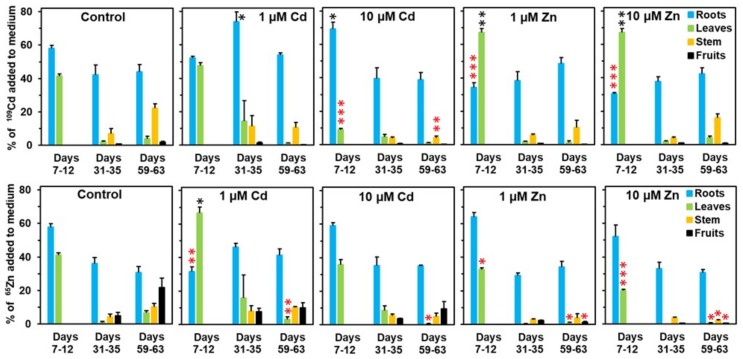
Influence of elevated concentrations in the nutrient medium on the distribution of ^109^Cd and ^65^Zn. Young plants (10 d old) were transferred at day 0 to the nutrient media with different cadmium and zinc concentrations. The labeling period is indicated below the diagrams: the first number indicates the beginning (addition of ^109^Cd and ^65^Zn to the nutrient media) and the second number the end (plant analysis) of the labeling phase. Means + SE of four replicates are shown. Significantly higher (*, **, ***) and lower (*, **, ***) values than in control plants on the *P* = 0.05 (*, *), *P* = 0.01 (**, **), and *P* = 0.001 (***, ***) level are indicated above the columns (Student’s t-test).

In a first set of experiments (Figure 1), plants were grown on unlabeled nutrient media with various zinc or cadmium concentrations and the radionuclides ^109^Cd and ^65^Zn were added to the nutrient media at day 7, day 31, or day 59. The plants were collected 4 or 5 days after adding ^109^Cd and ^65^Zn and the radiolabels were detected in the separated organs by gamma spectrometry (Figure 1). The data from these analyses refer to the distribution of cadmium and zinc recently taken up from the nutrient media. In controls, 40%–50% of ^109^Cd and ^65^Zn added to the medium reached the aerial plant parts. A considerable portion of the ^109^Cd (but not the ^65^Zn) accumulated in the stem. The transport of ^109^Cd to the shoot was stimulated by increased zinc in the medium in young plants and was ineffective in older plants. No significant effect on ^109^Cd transport was observed for 1 µM Cd, while transfer to the shoot was drastically reduced for 10 µM Cd. In contrast, ^65^Zn transport to the shoot was only moderately affected by 1 or 10 µM Cd, but was drastically decreased by 1 or 10 µM Zn in the medium. It should be noticed that the radioisotope contents are expressed as the % of the label added to the medium and the sum in the various plant parts may therefore be less than 100%. Parts of the label may not have been taken up, were released again from roots into the medium, or were still present in senesced leaves falling from the plants. The results from these analyses indicate that the effects were rather specific and not general effects on transport.

**Figure 2:**
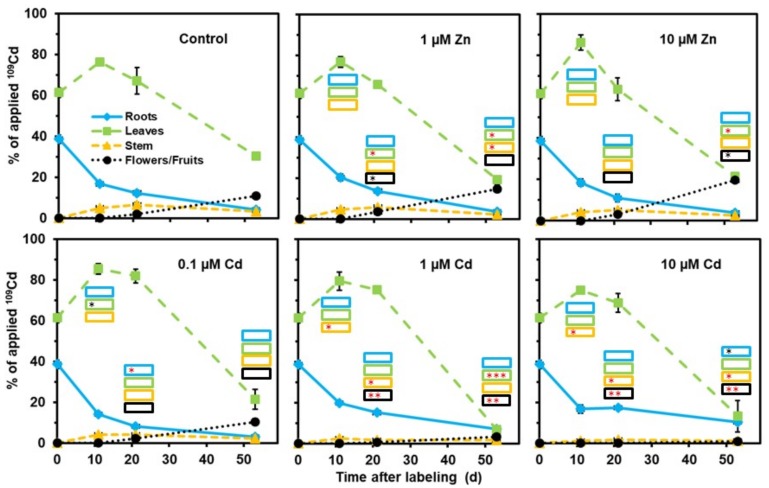
Influence of elevated cadmium (1 µM Cd and 10 µM Cd) or zinc (1 µM Zn and 10 µM Zn) concentrations in the nutrient medium on the redistribution of ^109^Cd. The plants were labeled with ^109^Cd in standard nutrient medium for 3 d before transferring them to the media indicated (day 0). Means + SE of four replicates are shown. Significantly higher (*, **, ***) and lower (*, **, ***) values than in control plants on the *P* = 0.05 (*, *), *P* = 0.01 (**, **), and *P* = 0.001 (***, ***) level are indicated in the insets for the various plant parts collected on day 11, day 21, and day 53 (Student’s t-test).

**Figure 3:**
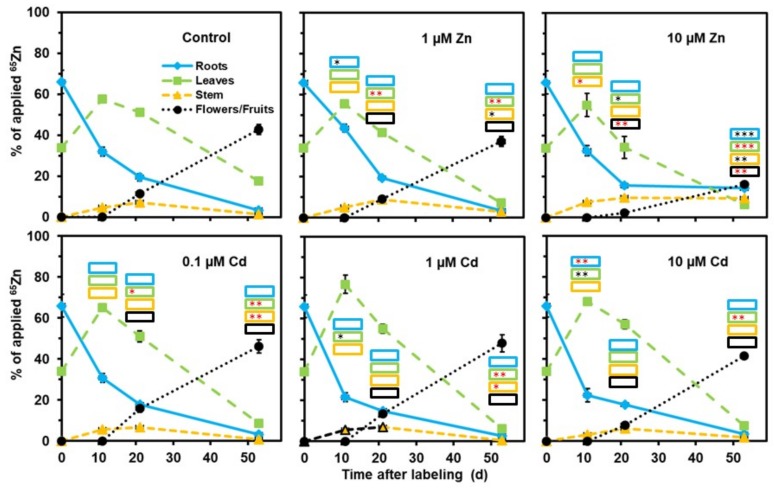
Influence of elevated cadmium (1 µM Cd and 10 µM Cd) or zinc (1 µM Zn and 10 µM Zn) concentrations in the nutrient medium on the redistribution of ^65^Zn. The plants were labeled with ^65^Zn in standard nutrient medium for 3 d before transferring them to the media indicated (day 0). Means + SE of four replicates are shown. Significantly higher (*, **, ***) and lower (*, **, ***) values than in control plants on the *P* = 0.05 (*, *), *P* = 0.01 (**, **), and *P* = 0.001 (***, ***) level are indicated in the insets for the various plant parts collected on day 11, day 21, and day 53 (Student’s t-test).

In a second series of experiments, young plants (grown on standard nutrient medium) were labeled 17 d after initiating germination with ^109^Cd and ^65^Zn for 3 d and the labeled plants were then transferred to the various media. This setup was very helpful for addressing the redistribution processes within the shoot throughout growth and maturation. Increased zinc concentrations in the medium caused no major effect on ^109^Cd redistribution, while 1 or 10 µM (but not 0.1 µM) cadmium in the medium negatively influenced the root-to-shoot transfer and the redistribution from vegetative shoot parts to the reproductive organs (Figure 2). The decrease in the leaves was mainly caused by the loss of leaf biomass during reproductive growth. In general, ^65^Zn was more efficiently transferred to flowers and fruits than ^109^Cd (Figure 2 and Figure 3). No major effect of cadmium or 1 µM Zn on the distribution of ^65^Zn between roots, the stem, leaves, and flowers/fruits was visible, but 10 µM Zn in the medium clearly lowered the release from the roots to the shoot and the subsequent transfer to the reproductive organs (Figure 3). The still relatively high level of ^65^Zn in the stem of plants for 10 µM Zn at day 53 may be due to a retention of ^65^Zn delivered from the roots via the xylem to the shoot or of ^65^Zn remobilized via the phloem from leaves (Figure 3).

**Figure 4:**
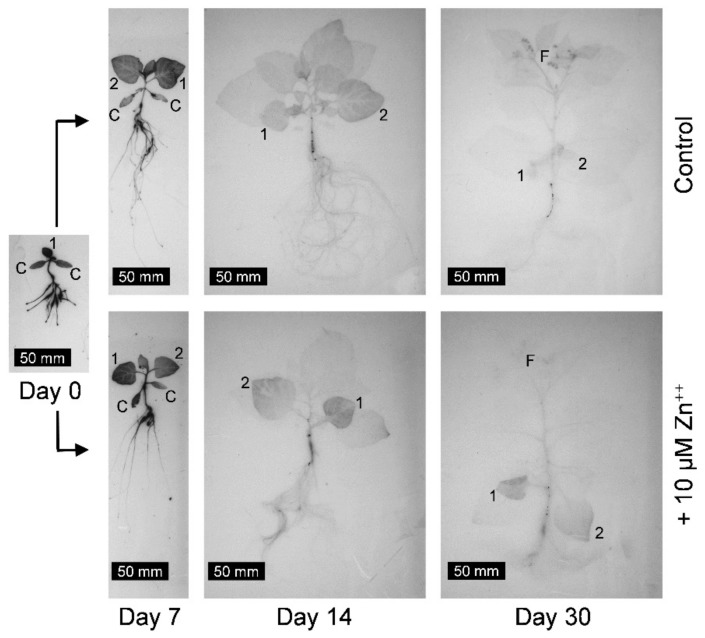
Autoradiographs of plants labeled for 1 d with ^65^Zn before day 0 and then incubated for 7, 14, or 30 d on standard medium without (Control) or with additional zinc (+10 µM Zn^++^). Cotyledons (C), leaf 1 (1), leaf 2 (2), and flowers/fruits (F) are indicated.

This effect was visualized by autoradiography (Figure 4). In control plants, the label moved from roots to older leaves, then to younger leaves, and finally to flowers/fruits. In the presence of 10 µM Zn, the label remained in leaves 1 and 2, while only trace amounts reached younger leaves and flowers/fruits.

**Figure 5:**
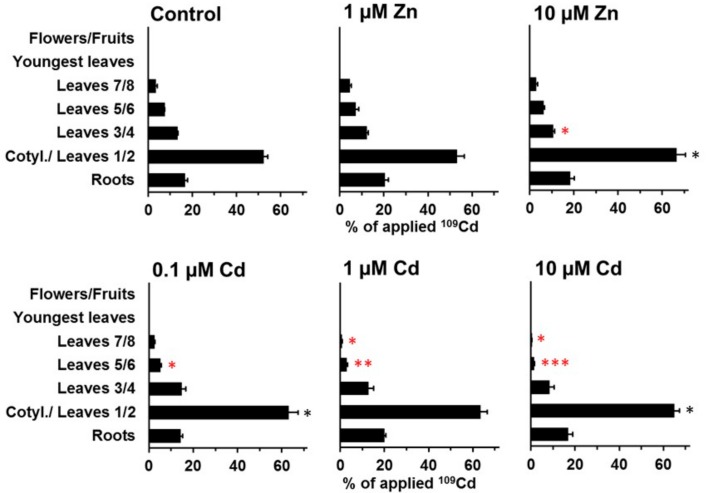
Redistribution of ^109^Cd in plants grown on standard nutrient medium (Control) or on the same medium with increased Zn (1 µM Zn and 10 µM Zn) or Cd (0.1 µM Cd, 1 µM Cd, and 10 µM Cd) concentrations for 11 d after the labeling phase (duration: 3 d). Means + SE of four replicates are shown. Significantly higher (*, **, ***) and lower (*, **, ***) values than in control plants on the P = 0.05 (*, *), P = 0.01 (**, **), and P = 0.001 (***, ***) level are indicated (Student’s t-test).

**Figure 6:**
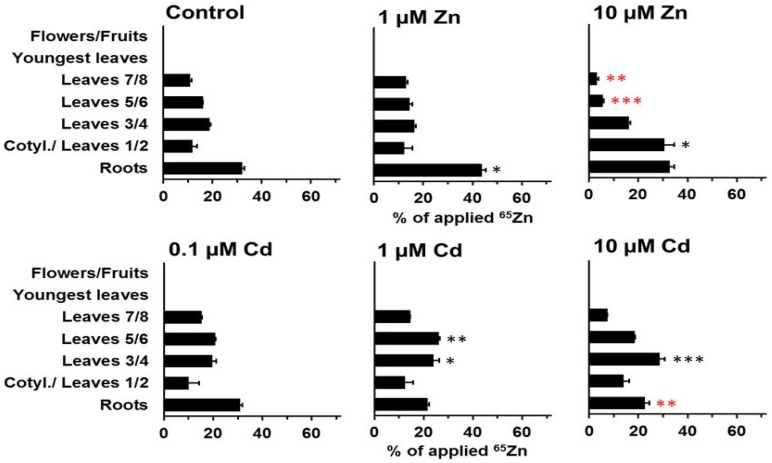
Redistribution of ^65^Zn in plants grown on standard nutrient medium (Control) or on the same medium with increased Zn (1 µM Zn and 10 µM Zn) or Cd (0.1 µM Cd, 1 µM Cd, and 10 µM Cd) concentrations during 11 d after the labeling phase (duration: 3 d). Means + SE of four replicates are shown. Significantly higher (*, **, ***) and lower (*, **, ***) values than in control plants on the P = 0.05 (*, *), P = 0.01 (**, **), and P = 0.001 (***, ***) level are indicated (Student’s t-test).

**Figure 7:**
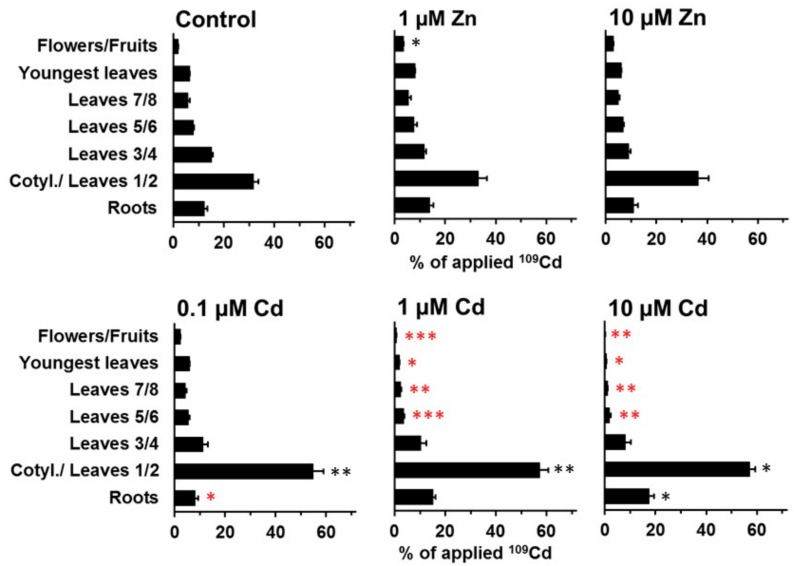
Redistribution of ^109^Cd in plants grown on standard nutrient medium (Control) or on the same medium with increased Zn (1 µM Zn and 10 µM Zn) or Cd (0.1 µM Cd, 1 µM Cd, and 10 µM Cd) concentrations for 21 d after the labeling phase (duration: 3 d). Means + SE of four replicates are shown. Significantly higher (*, **, ***) and lower (*, **, ***) values than in control plants on the P = 0.05 (*, *), P = 0.01 (**, **), and P = 0.001 (***, ***) level are indicated (Student’s t-test).

**Figure 8:**
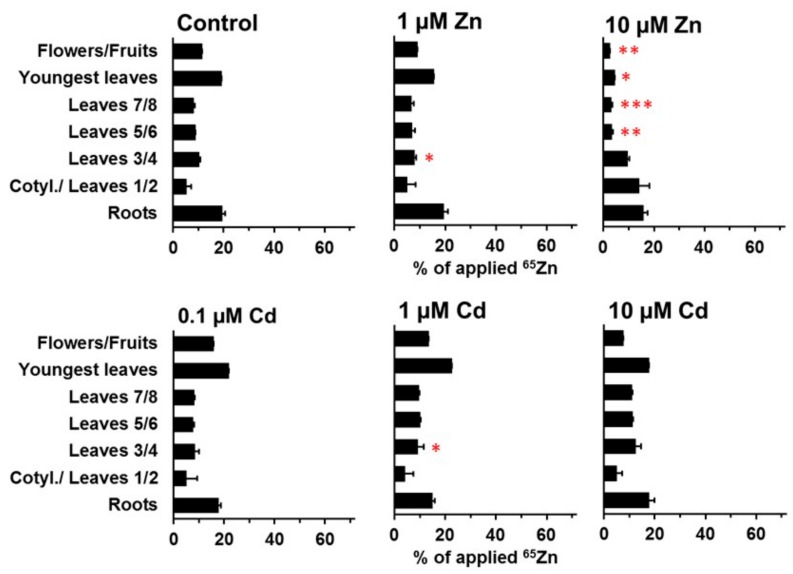
Redistribution of ^65^Zn in plants grown on standard nutrient medium (Control) or on the same medium with increased Zn (1 µM Zn and 10 µM Zn) or Cd (0.1 µM Cd, 1 µM Cd, and 10 µM Cd) concentrations for 21 d after the labeling phase (duration: 3 d). Means + SE of four replicates are shown. Significantly higher (*, **, ***) and lower (*, **, ***) values than in control plants on the P = 0.05 (*, *), P = 0.01 (**, **), and P = 0.001 (***, ***) level are indicated (Student’s t-test).

The separate analysis of leaves of different ages led to a more detailed picture of the redistribution within vegetative shoot parts (Figure 5, Figure 6, Figure 7 and Figure 8). At day 11, ^109^Cd was mainly (>50%) located in the cotyledons and leaves 1 and 2 (Figure 5). Zn addition to the medium caused no major differences in the ^109^Cd distribution within the shoot, while cadmium addition significantly decreased the transfer to the youngest leaves (leaves 5/6 and 7/8) in a consistent manner, from 0.1 to 10 µM Cd in the medium (Figure 5). The distribution of ^65^Zn considerably differed at day 11 from that of ^109^Cd (Figure 5 and Figure 6). Rather low ^65^Zn levels were detected at day 11 in cotyledons and leaves 1 and 2 (Figure 6). However, in the presence of 10 µM Zn in the medium, more ^65^Zn was retained in this sample and less ^65^Zn reached the top leaves. Some differences between the control and plants on 1 and 10 µM Cd were significant, but the overall patterns were still very similar and not comparable with the strong effect of 10 µM Zn.

The specific effects of zinc and cadmium in the medium on ^109^Cd and ^65^Zn distribution were far more pronounced at day 21 than at day 11 (Figure 5, Figure 6, Figure 7 and Figure 8). More leaves (“youngest leaves”) and flowers/fruits were also developed at day 21 and were included in the analysis as separate samples (Figure 7 and Figure 8). The decrease in ^109^Cd contents in cotyledons and leaves 1 and 2 between day 11 and day 21 was not significantly affected by elevated zinc concentrations in the medium (Figure 7). The transfer of ^109^Cd to the uppermost shoot parts (youngest leaves and flowers/fruits) was also not clearly influenced by zinc. ^109^Cd remobilization from the oldest leaves was negatively affected by cadmium in the medium (at all cadmium concentrations used). The transfer of ^109^Cd was significantly lower in the presence of 1 or 10 µM Cd than in controls or in the presence of high zinc levels or 0.1 µM Cd in the medium (Figure 7). The results from the same experimental setup with ^65^Zn are shown in Figure 8 and clearly illustrate the high specificity of cadmium and zinc influences on long-distance transport in *Solanum nigrum* L. No or only minor effects on ^65^Zn redistribution were observed in plants grown on nutrient solutions with cadmium (0.1, 1, and 10 µM Cd) or on a solution with 1 µM Zn. A strong inhibition of ^65^Zn redistribution from older leaves to the youngest leaves and to the reproductive structure was observed in plants grown on a nutrient solution with 10 µM Zn (Figure 8). The ^65^Zn content in cotyledons and leaves 1/2 was still high in these plants, but the difference in comparison to the control was not significant (large standard error). The negative effects of 10 µM Zn in the nutrient solution on the redistribution of ^65^Zn to the youngest leaves are highly significant and very visible in Figure 8. All of the results reported above document the generally specific effects of zinc and cadmium on the long-distance transport and final accumulation of the radioisotopes ^109^Cd and ^65^Zn.

## 3. Discussion

The previously reported efficient transfer of cadmium from the roots to the shoot in *Solanum nigrum* L. was confirmed in this study and further investigated considering the effects of increased cadmium or zinc concentrations on redistribution processes for these two heavy metals. The findings from the experiments reported here document that cadmium is even more rapidly transferred to the shoot than zinc. However, further redistribution within the aerial parts clearly differs for the two heavy metals. While more than 40% of the initially introduced ^65^Zn may finally reach the flowers/fruits, this value is far lower for ^109^Cd. The separate analyses of the contents in various leaves document a strong retention of ^109^Cd in the oldest leaves, which is most likely due to a poor redistribution via the phloem. Phloem loading must be considered as the key process for the redistribution within the shoot. The cause for poor remobilization may be either a strong retention of cadmium in leaf cells or in the leaf apoplast, preventing the heavy metals from reaching the phloem or the transfer into the phloem itself. The findings reported here indicate that the effects of increased cadmium or zinc levels quite selectively affect the redistribution of the two heavy metals from older leaves to younger leaves and to reproductive organs. Therefore, it appears unlikely that general effects (e.g., competition for endogenous chelators and toxic effects on metabolism) were the main mechanisms influencing cadmium and zinc redistribution in the shoots of *Solanum nigrum*.

The content of cadmium in the whole shoot may considerably decrease during maturation, since senesced leaves still containing this heavy metal may be lost. This effect is far less important for zinc than for cadmium, since zinc was more efficiently transported from older leaves to younger leaves and to flowers/fruits. Two important processes must therefore be distinguished: (a) the acropetal transport from the roots to transpiring parts of the shoot via the xylem and (b) the further redistribution via the phloem. Leaf senescence and the content of heavy metals in fallen leaves are important when establishing remediation protocols and the time for removing biomass from contaminated fields. The points mentioned above are highly relevant when considering a plant for bioremediation and for planning the bioremediation (especially plant parts to be collected and timing of collection). Such aspects must also be borne in mind for the selection or breeding of suitable genotypes for bioremediation.

The physiological processes involved in the rapid root-to-shoot transfer of cadmium in *Solanum nigrum* L. remain to be elucidated in more detail. The compartmentation on the tissue and on the subcellular level is an especially open question and might be addressed with suitable techniques. Chelators may also play a key role in this context, but so far, essentially no information is available for this plant. Cobalt was only very slowly transported from the roots to the shoot [8], indicating that the efficient root-to-shoot transfer observed for cadmium is a rather specific, and not general, phenomenon.

## 4. Materials and Methods

The source of the seeds and plant cultivation [8] were published earlier in detail and are therefore only summarized here. Dry *Solanum nigrum* L. seeds were soaked for 1 d at 4 °C in 0.1% (w/v) KNO_3_, germinated for 7 d on tissue paper moistened with 0.1% (w/v) KNO_3_, and afterwards grown hydroponically on a standard nutrient medium, as reported previously [8]. The standard medium contained 6 mM KH_2_PO_4_, 3 mM MgSO_4_, 1.36 mM Ca(NO_3_)_2_, 0.88 mM KNO_3_, 30 µM Fe as Fe-EDDHA, 1 µM MnCl_2_, 5 µM, H_3_BO_3_, 0.16 µM ZnSO_4_, 0.2 µM Na_2_MoO_4_, 0,048 µM Ni(NO_3_)_2_, and 0.1 µM CuSO_4_ [8]. The pH was in the range of 4.4–4.7. Cadmium was not added to a standard nutrient medium, but was present as a contaminant in solutes added and was detected in a concentration of 0.0016 µM. To prepare nutrient media with increased zinc or cadmium concentrations, ZnCl_2_ or CdCl_2_ (Sigma-Aldrich, Buchs, SG, Switzerland) were added from 100 mM stock solutions to a standard nutrient medium. All experiments were performed in a culture room with 14 h light (200 µmol photons m^−2^ s^−1^ from 4 Lumilux, Osram FQ 39 W/840 HO and 4 Lumilux, Osram FQ 39 W/830 HO) and 10 h darkness. The room temperature was 25 °C during the light phase and 20 °C during the dark phase.

Two types of experiments were performed. For the experiment documented in Figure 1, young plants (10 d old) were transferred to a standard medium or to a standard medium with an increased Cd (+1 µM Cd or +10 µM Cd) or increased zinc concentration (+1 µM Zn or +10 µM Zn). From preliminary experiments, it became evident that the growth of *Solanum nigrum* is severely affected during long-term exposure to cadmium concentrations above 10 µM, while higher zinc concentrations caused no visible symptoms. Based on these observations, the highest concentration used in the experiments reported here was 10 µM for both elements in the nutrient medium. Details of the timing of the various labeling phases are indicated in the legend of Figure 1. The plants were transferred individually to containers with labeling solution containing ^109^Cd (from Amersham BioSciences UK Ltd., Chalfont St. Giles, Buckinghamshire, United Kingdom) and ^65^Zn (from PerkinElmer AG, Schwerzenbach, ZH, Switzerland) to avoid competition between plants for the radionuclides and to achieve well-standardized labeling of the plants. The zinc and cadmium concentrations during the labeling phase were identical to those before labeling. Since the plants were rapidly growing, it was necessary to adapt the volume of the labeling solution for the three labeling periods. For the labeling phase from day 7 to day 12, each plant was supplied with 20.5 mL of the solution indicated (containing 3.1 Bq ^109^Cd and 3.1 Bq ^65^Zn), for the phase from day 31 to day 35 with 151 mL (containing 6.2 Bq ^109^Cd and 6.2 Bq ^65^Zn), and for the labeling phase from day 59 to day 63 with 242 mL (containing 12.4 Bq ^109^Cd and 12.3 Bq ^65^Zn). After the labeling phase of 4 or 5 days, plants (four replicates per treatment) were collected and dissected and the separated organs were dried prior to analyzing the ^109^Cd and ^65^Zn contents by gamma spectrometry. After completing the experiment, all samples were analyzed with a gamma counter (1480 Wizard 3′, Wallac Oy, Turku, Finland), allowing the simultaneous measurement of ^109^Cd and ^65^Zn and equipped with automatic correction for decay with the half-life. To allow a comparison of the two isotopes and the three labeling intervals, the contents in the various plant parts were expressed as the % of the label added to the nutrient medium for each plant.

For another series of analyses (Figure 2, Figure 3, Figure 5, Figure 6, Figure 7 and Figure 8), seeds were germinated on moistened tissue paper and the young plants (10 d old) were grown on a standard nutrient solution, as mentioned above. Plants (17 d old) were then individually transferred to small bottles with 20.5 mL standard nutrient solution containing 3.1 Bq ^109^Cd and 3.1 Bq ^65^Zn (but without increased cadmium or zinc concentrations). After the labeling phase (3 d), the plants were placed in darkened containers with the various media (standard nutrient medium; standard nutrient with 0.1, 1, or 10 µM Cd; standard nutrient with 1 or 10 µM Zn). Nutrient media were replaced with fresh media containing the same concentrations of heavy metals repeatedly throughout plant growth and maturation. Plants (four replicates per treatment) were collected at day 0 (immediately after the labeling phase), day 11, day 21, and day 53. The various organs were immediately separated and dried prior to analyzing the ^109^Cd and ^65^Zn contents by gamma spectrometry, as mentioned above.

Plant preparation for autoradiography (Figure 4) was described previously [18]. Only one radionuclide can be applied to a plant, since proper discrimination is not possible (in contrast to gamma spectrometry). Young plants (13 d old) were labeled separately for one day on tubes with 3 mL standard nutrient solution containing 18.6 Bq ^65^Zn. After the labeling phase (day 0), the plants were transferred to the nutrient medium indicated (without the radionuclide). Plants were collected at day 0, day 7, day 14, and day 30. The plants were prepared immediately after collection for autoradiography, dried, and then stored and exposed simultaneously at the end of the experiment for 227 d to X-ray films (Fuji medical X-ray film, super RX, Fujifilm AG, Dielsdorf, ZH, Switzerland). The long exposure time was necessary to compensate for the low ^65^Zn contents in the plants. For technical reasons (no suitably labeled plants available), autoradiography was not possible for ^109^Cd.
